# MicroRNA-22 Promotes Renal Tubulointerstitial Fibrosis by Targeting PTEN and Suppressing Autophagy in Diabetic Nephropathy

**DOI:** 10.1155/2018/4728645

**Published:** 2018-04-03

**Authors:** Yingying Zhang, Siqi Zhao, Depei Wu, Xingmei Liu, Mingjun Shi, Yuanyuan Wang, Fan Zhang, Jing Ding, Ying Xiao, Bing Guo

**Affiliations:** ^1^Department of Pathophysiology, Guizhou Medical University, Guiyang, Guizhou 550025, China; ^2^Laboratory of Pathogenesis Research, Drug Prevention and Treatment of Major Diseases, Guizhou Medical University, Guiyang, Guizhou 550025, China

## Abstract

Renal tubulointerstitial fibrosis (TIF) is a major feature of diabetic nephropathy (DN). There is increasing evidence demonstrating that microRNAs act as key players in the regulation of autophagy and are involved in DN. However, the exact link among microRNAs, autophagy, and TIF in DN is largely unknown. In this study, our results showed that TIF was observed in DN rats together with obvious autophagy suppression. Moreover, microRNA-22 (miR-22) was upregulated and associated with reduced expression of its target gene phosphatase and tensin homolog (PTEN) in both the kidneys of DN rats and high glucose-cultured NRK-52E cells. Intriguingly, induction of autophagy by rapamycin antagonized high glucose-induced collagen IV (Col IV) and *α*-SMA expression. In addition, ectopic expression of miR-22 suppressed autophagic flux and induced the expression of Col IV and *α*-SMA, whereas the inhibition of endogenous miR-22 effectively relieved high glucose-induced autophagy suppression and the expression of Col IV and *α*-SMA in NRK-52E cells. Overexpression of PTEN protectively antagonized high glucose- and miR-22-induced autophagy suppression and the expression of Col IV. Therefore, our findings indicated that miR-22 may promote TIF by suppressing autophagy partially via targeting PTEN and represents a novel and promising therapeutic target for DN.

## 1. Introduction

Renal tubulointerstitial fibrosis (TIF) frequently occurs in diabetic nephropathy (DN), which is a main cause of end-stage renal disease throughout the world [1, 2]. The degree of TIF is associated with declined renal function and it is characterized by tubular atrophy and excessive accumulation of extracellular matrix components, such as collagens and fibronectin. However, the mechanism by which TIF develops in DN warrants exploration. Autophagy is an important homoeostatic process that mediates the degradation of damaged organelles or protein aggregates. Although recent studies suggest that activation of autophagy may be a protective measure to suppress TIF and prevent the progression of DN [3, 4], the event that initiates this process remains unclear.

Increasing evidence has demonstrated that phosphatase and tensin homolog (PTEN) is closely related to renal fibrosis in DN [5, 6]. This phosphatase acts as a negative regulator of fibrosis, such as cardiac fibrosis, lung fibrosis, and liver fibrosis [7–9]. It can attenuate renal fibrosis by inhibiting the synthesis and accumulation of matrix protein and interacting with TGF-*β* and members of the Akt pathway [10–12]. Moreover, PTEN is a key regulator of autophagy. It can activate autophagy in a number of different disease models or cell types by inhibiting the PI3K/Akt/mTOR signaling pathway [13]. Therefore, exploring the regulation of PTEN may be a significant way to restrain the development of TIF and DN.

MicroRNAs (miRNAs) are a group of highly conserved, small noncoding RNAs that can modulate gene expression through posttranscriptional binding to the 3′UTR of their target genes [14]. They play an important role in autophagy and various pathological processes including diabetes and fibrosis [15–17]. Recently, the emerging roles of miRNAs in the pathogenesis of DN have been highlighted [18, 19]. However, to date, few miRNAs have been found to execute their biological functions in DN by targeting PTEN, such as miR-21, miR-214, and miR-217 [20–22]. Meanwhile, only a few miRNAs have been found to have an effect on DN by regulating autophagy [22]. Thus, it is becoming clear that miRNAs with the ability to regulate PTEN and autophagy may become a promising therapeutic target for DN.

In this study, we demonstrated that miR-22 promoted the expression of collagen IV (Col IV) and *α*-SMA by regulating the PTEN/Akt/mTOR pathway and suppressing autophagy in NRK-52E cells. Inhibition of the endogenous miR-22 increased autophagy and alleviated high glucose- (HG-) induced Col IV and *α*-SMA expression. Therefore, our results suggested that miR-22 might be a novel target for the treatment of TIF and DN.

## 2. Materials and Methods

### 2.1. Experimental Animals

Twenty healthy and specific pathogen-free (SPF) male Sprague-Dawley rats weighing 180 ± 20 g were provided by Beijing HFK Bioscience Co. Ltd. (Beijing, China). The animals were housed in a clean environment under a controlled temperature of 20–25°C in the animal center of Guizhou Medical University (Guizhou, China). All rats were randomly divided into a control group (NC group, *n* = 10) and a diabetic nephropathy group (DN group, *n* = 10). DN rats were induced by injecting streptozotocin (STZ; Sigma, MO, USA) in the tail vein at a dose of 55 mg/kg, while NC rats were injected with an equal volume of solvent. All rats were given a normal diet and unlimited drinking water. After 48 h, the high blood glucose level (fasting blood glucose ≥ 16.7 mM) indicated that the DN rat model was established successfully. Ten weeks after STZ or solvent injection, 24 h urine and femoral arterial blood from each rat was collected before the rats were sacrificed and urine protein and various biochemical indices were measured, respectively. All rats were anesthetized with intraperitoneal sodium pentobarbital (50 mg/kg) and were sacrificed by drawing-out all the blood from their hearts. The kidneys of each rat were harvested, one was fixed in 4% paraformaldehyde and the other one was immediately frozen in liquid nitrogen. The kidney index (KI) was calculated by right kidney weight (KW) and body weight (BW): KI = KW/BW. The study was conducted in accordance with the guidelines of the National Health and Medical Research Council of China's Code for the care and use of animals for scientific purpose.

### 2.2. Biochemical Assays

Blood glucose was measured by the oxidase method and 24 h urinary protein was measured by the Coomassie Brilliant Blue method. All tests were analyzed by a 1650 automatic biochemical analyzer (Beckman Instruments, CA, USA).

### 2.3. Cell Culture

NRK-52E cells were cultured in Dulbeccos's modified Eagle's medium (DMEM; Hyclone, UT, USA) supplemented with 5% fetal bovine serum (FBS; Gibco, CA, USA) containing normal glucose (NG, 5.5 mM glucose) or HG (30 mM glucose). Cells in each group were seeded in 25 cm^2^ culture flasks and cultured in the incubator with 5% CO_2_ at 37°C for 24 h or 48 h for further study.

### 2.4. Histology and Immunohistochemistry

Renal tissue paraffin sections were subjected to hematoxylin-eosin (HE) staining, and renal tissue fibrosis was observed by Masson's trichrome. The biotin-streptavidin-peroxidase method (ZSBIO, Beijing, China) was applied to conduct immunohistochemistry. The primary antibody against Col IV (SAB4200500) was purchased from Sigma. Primary antibody (1 : 100) was diluted in phosphate-buffered saline (PBS) containing 1% bovine serum albumin (BSA), and the secondary antibodies used were affinity-purified biotinylated goat antimouse IgG. Sections incubated with PBS served as the negative control. Two blinded investigators observed all slides independently, and fifty fields of view were analyzed by Image-Pro plus 6.0.

### 2.5. Real-Time PCR Detection

Total RNA was extracted from renal tissues of NC or DN rats and NRK-52E cells using the TRIzol reagent (Invitrogen, CA, USA) according to manufacturer's protocol. For quantification of miR-22, the Bulge-Loop™ miRNA qRT-PCR primer kits (RiboBio, Guangzhou, China) were utilized following manufacturer's instructions. qPCR was performed using SuperReal PreMix (SYBR Green) (Tiangen, Beijing, China) with iQ SYBR Green SuperMix (Bio-Rad). The expression of miR-22 was defined from the threshold cycle (Ct), and relative expression levels were calculated using the 2^−ΔΔCt^ method after normalization with reference to the expression of U6 snRNA.

### 2.6. Western Blotting

Renal tissues and NRK-52E cells were lysed in RIPA lysis buffer (50 mM Tris-HCl, pH 7.4, 150 mM NaCl, 1% Triton X-100, 1% sodium deoxycholate, 0.1% SDS), supplemented with a complete protease inhibitor mixture (P0013B, Beyotime Shanghai, China). Subsequently, 30 *μ*g protein samples were separated on an 8, 10, or 12.5% Tris-glycine gel and transferred onto a nitrocellulose membrane. The primary antibodies used for Western blotting were as follows: PTEN (Cell Signaling, 1 : 1000), Col IV (Sigma, 1 : 1000), LC3 (Cell Signaling, 1 : 1000), p62 (Cell Signaling, 1 : 2000), Akt (Cell Signaling, 1 : 1000), p-Akt (Cell Signaling, 1 : 1000), mTOR (Cell Signaling, 1 : 1000), p-mTOR (Cell Signaling, 1 : 1000), and *β*-actin (Actin; Abcam, 1 : 2000).

### 2.7. Luciferase Reporter Assays

The fragment of the predicted miR-22 binding sequence or a mismatch sequence in the 3′UTR of PTEN mRNA, amplified from 293T genomic DNA, was cloned into the SpeI and HandIII restriction sites of the pMIR-REPORT plasmid (Invitrogen). The predicted target site was mutated by site-directed mutagenesis, and 50 nM miR-22 mimics or NC mimics (RiboBio, Guangzhou, China) were transfected into NRK-52E cells with 5 ng Renilla plasmid (Promega, Wisconsin, USA) and 100 ng of the WT or MUT plasmid that contain the 3′UTR of PTEN. Twenty-four hours before transfection, 5 × 10^4^ cells were seeded in 24-well plates. A luciferase assay was performed 24 h after transfection using the dual-luciferase reporter assay system (Promega). The firefly luciferase activity was normalized to the Renilla luciferase activity.

### 2.8. Confocal Microscopy Analysis

NRK-52E cells were first transfected with pDsRed-LC3, and then they were transfected with NC mimics or miR-22 mimics for 24 h. Following this incubation, cells were cultured with rapamycin (Rap) and NG or HG for another 24 h. The cells were then fixed and immediately analyzed by confocal microscopy (Olympus, FV1000, Japan). RFP-LC3 puncta formation and the localization of RFP-LC3 were observed.

### 2.9. Statistical Analysis

All experiments were repeated at least three times. The statistical significance between two groups was assessed using the Student's 2-tailed *t*-test. One-way ANOVA followed by a Bonferroni-Dunn test was used for the comparison of more than two groups. Data were expressed as mean ± standard error of mean (SEM). *P* < 0.05 was considered significant.

## 3. Results

### 3.1. Decreased Autophagy Level Was Associated with Renal Fibrosis in DN Rats

**Figure 1:**
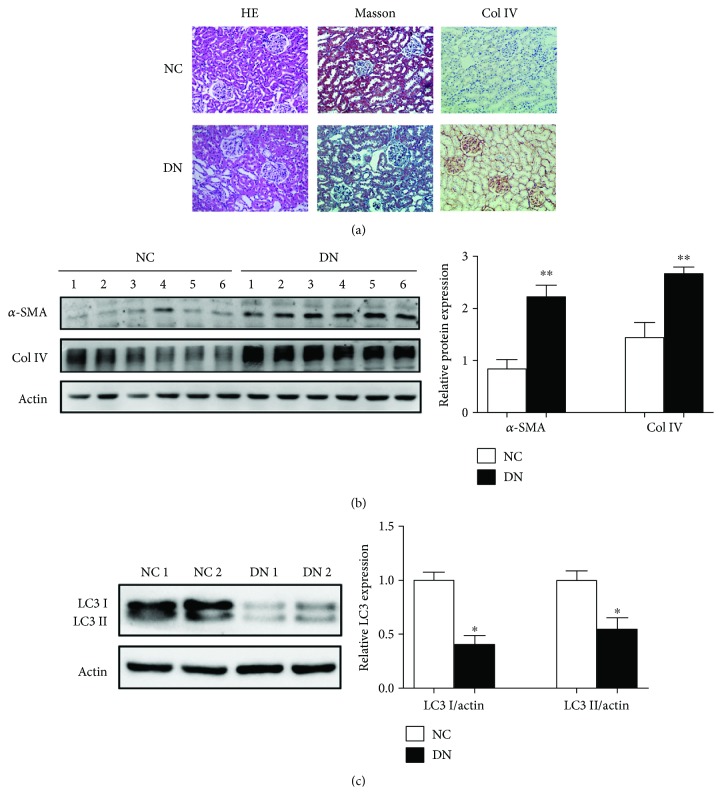
Developed renal fibrosis was accompanied by decreased autophagy in DN rats. (a) Representative photograph of histological changes and immunohistochemical staining for Col IV protein expression analysis of renal tissues in NC group and DN group. Figures were shown at ×200 magnification. For (b) and (c), Western blot assay was performed to detect the expression of Col IV, *α*-SMA, LC3-I, and LC3-II in renal tissues in NC group and DN group. ImageJ densitometric analysis of the level of Col IV, *α*-SMA, LC3-I, and LC3-II; data represent mean ± SEM, *n* = 10, ^∗^ *P* < 0.05 and ^∗∗^ *P* < 0.01.

In this study, our result showed that rats treated with STZ injection for 48 h presented a high blood glucose level. At the end of the study, KI, blood glucose, and 24 h urinary protein increased in DN rats as compared to healthy and SPF male Sprague-Dawley rats (Table 1). Histochemical staining was used to check and define the pathological changes in renal tissues. HE staining showed that the epithelial cells of the renal tubule emerged with mild vacuolar degeneration, and the renal tubules were distended with macrophages infiltrating the renal interstitium in the DN group (Figure 1(a)). The Masson stain revealed that the DN rats developed renal fibrosis and immunohistochemistry showed an upregulation of Col IV in renal tissues of DN rats under the light microscope (Figure 1(a)). Moreover, the expression of Col IV and *α*-SMA was upregulated, while LC-3I and LC-3II were decreased in renal tissues in DN group compared to control group as shown by Western blotting (Figures 1(b) and 1(c)).

### 3.2. Improving Autophagy Antagonized HG-Induced Col IV Expression

**Figure 2:**
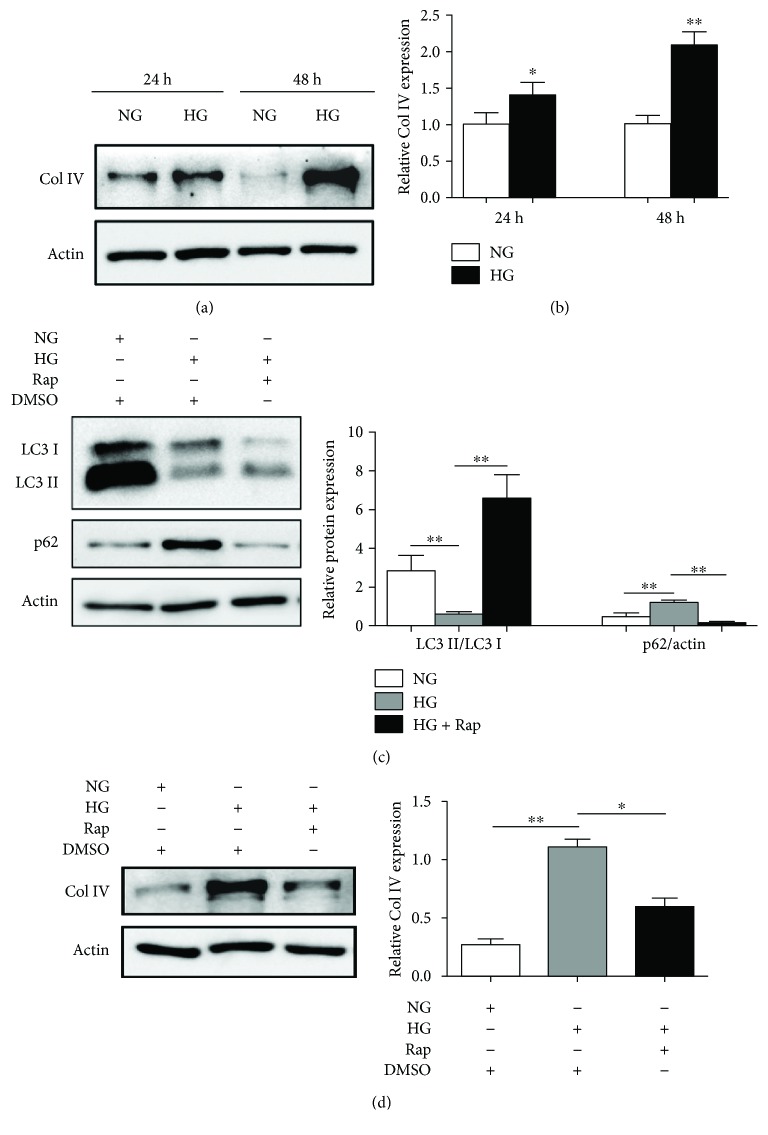
The function of autophagy on HG-induced expression of Col IV and *α*-SMA in NRK-52E cells. (a) NRK-52E cells were treated with NG or HG for 24 or 48 h; the protein level of Col IV was detected by Western blot. (b) ImageJ densitometric analysis of the protein level of Col IV; data represent mean ± SEM, *n* = 3, ^∗^ *P* < 0.05, ^∗∗^ *P* < 0.01. For (c) and (d), NRK-52E cells were treated with DMSO or 100 nM Rap and NG or HG for 48 h. The expression of LC3-I, LC3-II, p62, and Col IV was detected by Western blot. ImageJ densitometric analysis of the protein levels; data represent mean ± SEM, *n* = 3, ^∗^ *P* < 0.05, ^∗∗^ *P* < 0.01. Rap: rapamycin.

We next evaluate the effect of autophagy on the expression of Col IV *in vitro*. First of all, we used mannitol as a control, and the result showed that there had no obvious influence of mannitol on the expression of PTEN and *α*-SMA together with autophagy (Supplementary Figure 1). Western blots showed that Col IV levels were significantly increased after NRK-52E cells were treated with HG for 24 and 48 h as compared to the NG-treated group (Figures 2(a) and 2(b)). Then, NRK-52E cells were treated with DMSO or 100 nM Rap which is an autophagy agonist and NG or HG for 48 h. The conversion of LC3-I to LC3-II and the degradation of p62 were inhibited, and the expression of Col IV was upregulated after cells were treated with HG for 48 h as compared to the NG-treated group (Figures 2(c) and 2(d)). Moreover, as shown in Figures 2(c) and 2(d), Rap could effectively improve the conversion of LC3-I to LC3-II and the degradation of p62 and suppress the expression of Col IV. Therefore, autophagy may be a protective factor that inhibits the synthesis of Col IV and *α*-SMA, and thus, antagonizes TIF.

### 3.3. MiR-22 Was Upregulated and PTEN Was Downregulated in Renal Tissues of DN Rats and HG-Treated NRK-52E Cells

**Figure 3:**
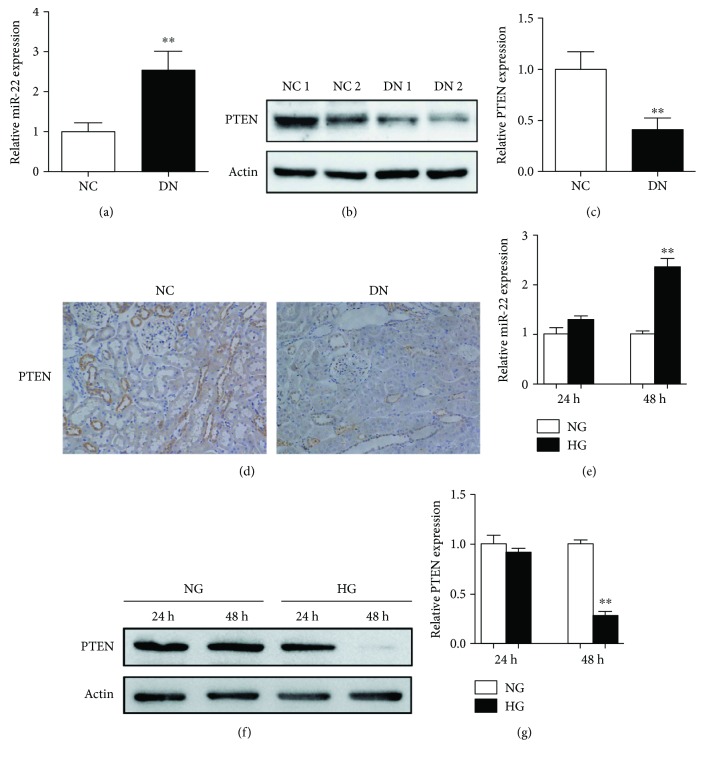
MiR-22 upregulated was associated with reduced PTEN in DN in vivo and in vitro. (a) The expression of miR-22 in renal tissues of NC group and DN group was analyzed by qPCR; data represent mean ± SEM, *n* = 10, ^∗∗^ *P* < 0.01. (b) The protein level of PTEN in renal tissues of NC group and DN group was detected by Western blot (c) and analyzed by ImageJ; data represent mean ± SEM, *n* = 10, ^∗∗^ *P* < 0.01. (d) Representative photograph of immunohistochemical staining for PTEN protein expression analysis of renal tissues in NC group and DN group. Figures were shown at ×200 magnification. (e) NRK-52E cells were treated with NG or HG for 24 or 48 h. The expression of miR-22 was analyzed by qPCR; data represent mean ± SEM, *n* = 3, ^∗∗^ *P* < 0.01. (f) The expression of PTEN was detected by Western blot (g) and analyzed by ImageJ; data represent mean ± SEM, *n* = 3, ^∗∗^ *P* < 0.01.

We further detected the expression of miR-22 and PTEN in renal tissues. The results showed a significantly increased expression of miR-22 and decreased expression of PTEN in the DN group as compared to the control group (Figure 3(a)–3(c)). In addition, immunohistochemistry indicated that the expression of PTEN was downregulated in renal tubule tissues of DN rats (Figure 3(d)). Subsequently, NRK-52E cells were cultured in NG or HG conditions for 24 and 48 h. Consistent with the results *in vivo*, the expression of miR-22 was upregulated and the protein level of PTEN was downregulated after HG treatment for 48 h, but there was no obvious differences in miR-22 and PTEN between NG- and HG-treated cells after 24 h (Figure 3(e)–3(g)).

### 3.4. PTEN Was a Direct Target of miR-22

**Figure 4:**
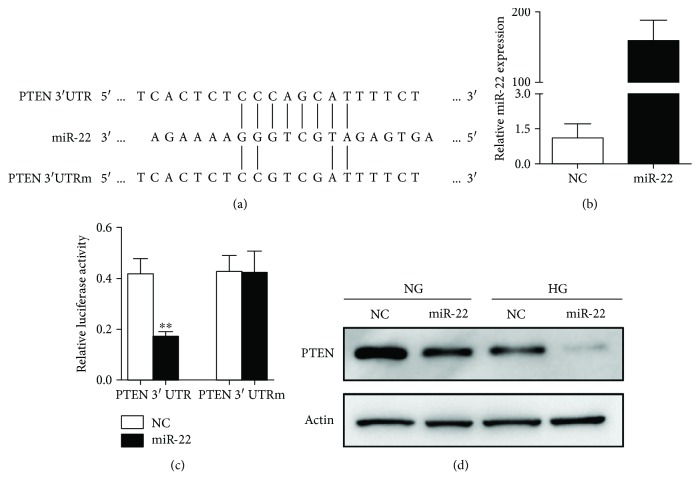
MiR-22 directly targets PTEN. (a) Predicted binding sequences and mutated site between miR-22 and seed site in 3′UTR of PTEN. (b) NRK-52E cells were transfected with miR-22 mimics or NC mimics for 24 h. qPCR was performed to detect the transfection efficiency of miR-22 mimics. (c) NRK-52E cells were cotransfected with 50 nM miR-22 mimics or NC mimics, 100 ng of the WT or MUT plasmid that contain 3′UTR of PTEN, and 5 ng Renilla plasmid; data represent mean ± SEM, *n* = 3, ^∗∗^ *P* < 0.01. (d) After transfected with miR-22 mimics or NC mimics for 24 h, NRK-52E cells were treated with NG or HG for another 48 h. The expression of PTEN was detected by Western blot.

For the dysregulation of miR-22 and PTEN in DN, we further explored their relationship in NRK-52E cells. By using TargetScan and miRanda bioinformatics tools, we found that miR-22 potentially targets PTEN (Figure 4(a)). Subsequently, we designed pMIR-reporter constructs containing either wild-type (WT) or mutated-type (Mut) miR-22 binding sites at the 3′UTR of PTEN. The expression of miR-22 was present at a high level after cells were transfected with miR-22 mimics for 24 h (Figure 4(b)). The luciferase reporter assay was performed 24 h after cotransfection with the WT or Mut PTEN 3′UTR clone and miR-22 or NC mimics. As shown in Figure 4(c), miR-22 mimics significantly reduced the luciferase activity of WT PTEN 3′UTR, but had no significant effect on Mut PTEN 3′UTR. Moreover, the protein expression of PTEN was downregulated by miR-22 after either NG or HG treatment for 48 h as compared with the negative controls in NRK-52E cells (Figure 4(d)).

### 3.5. miR-22 Inhibited Autophagy and Promoted the Expression of Col IV and *α*-SMA

**Figure 5:**
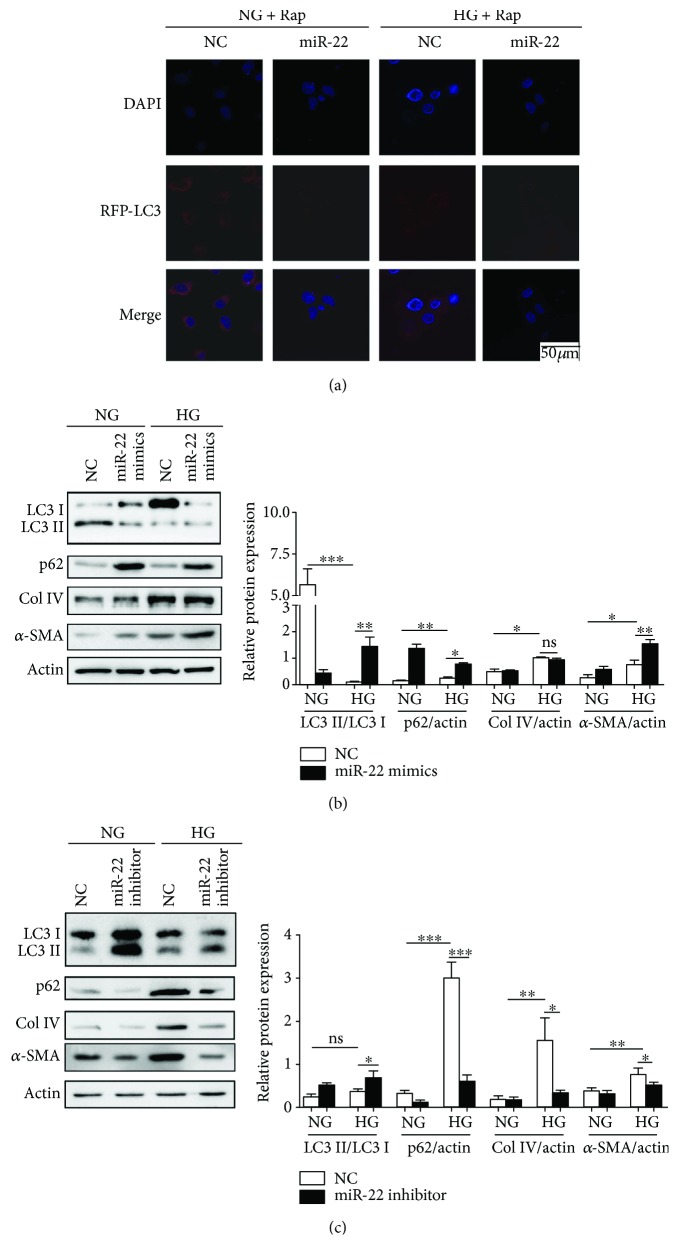
MiR-22 inhibited autophagy and mediated HG-induced Col IV and *α*-SMA expression. (a) After cotransfected with pDsRed-LC3 plasmid and miR-22 mimics or NC mimics for 24 h, NRK-52E cells were treated with NG or HG for another 48 h. NRK-52E cells were subsequently imaged using confocal microscopy. Scale bars represent 50 *μ*m. Rap: rapamycin. For (b) and (c), after transfected with miR-22 mimics or NC mimics or miR-22 inhibitor or NC inhibitor for 24 h, NRK-52E cells were treated with NG or HG for another 48 h. The expression of LC3-I, LC3-II, p62, Col IV, and *α*-SMA was detected by Western blot. ImageJ densitometric analysis of the protein levels; data represent mean ± SEM, *n* = 3, ^∗^ *P* < 0.05, ^∗∗^ *P* < 0.01, ^∗∗∗^ *P* < 0.001, ns: not significant.

We next determined whether miR-22 had an effect on autophagy and fibrosis. pDsRed-LC3 plasmid and miR-22 or NC mimics were cotransfected into NRK-52E cells for 24 h, and then cells were treated with NG or HG for another 48 h. Confocal microscopy was used to analyze the samples, and the results showed decreased accumulation of pDsRed-LC3 puncta in the cytoplasm of miR-22 mimics-transfected and/or HG-treated cells (Figure 5(a)). Western blots revealed that miR-22 could suppress the conversion of LC3-I to LC3-II and the degradation of p62, meanwhile it promoted the expression of Col IV and *α*-SMA in NG- or HG-treated NRK-52E cells (Figure 5(b)). Moreover, a miR-22 inhibitor improved autophagic flux and suppressed the expression of Col IV and *α*-SMA as compared to the negative control (Figure 5(c)). Thus, inhibition of miR-22 may suppress TIF by the induction of autophagy.

### 3.6. miR-22 Regulation of Autophagy and the Expression of Col IV Were Partially through Targeting PTEN

**Figure 6:**
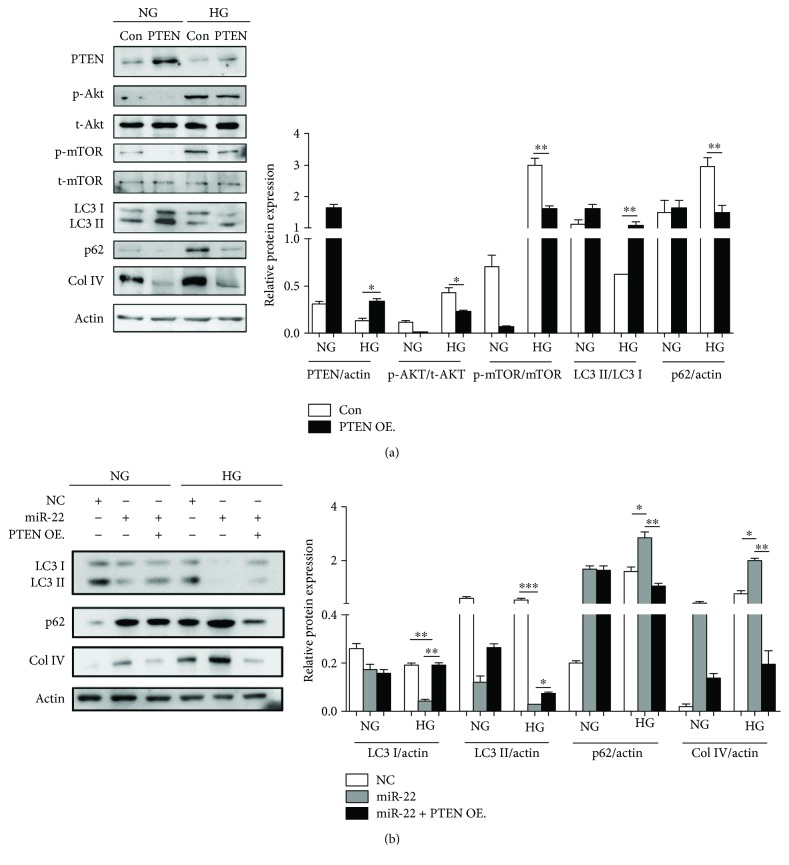
PTEN partially mediated the biological effect of miR-22 on autophagy and the synthesis of Col IV. (a) After transfected with PTEN overexpression plasmid or empty plasmid for 24 h, NRK-52E cells were cultured with NG or HG for another 48 h. The expression of PTEN, p-Akt, t-Akt, p-mTOR, t-mTOR, LC3-I, LC3-II, p62, and Col IV was detected by Western blot. p-Akt: phosphorylated Akt; t-Akt: total Akt; p-mTOR: phosphorylated mTOR; t-mTOR: total mTOR. ImageJ densitometric analysis of the protein levels; data represent mean ± SEM, *n* = 3, ^∗^ *P* < 0.05, ^∗∗^ *P* < 0.01. (b) After cotransfected with PTEN overexpression plasmid or empty plasmid and miR-22 mimics or NC mimics for 24 h, NRK-52E cells were treated with NG or HG for another 48 h. The expression of LC3-I, LC3-II, p62, and Col IV was detected by Western blot. ImageJ densitometric analysis of the protein levels; data represent mean ± SEM, *n* = 3, ^∗^ *P* < 0.05, ^∗∗^ *P* < 0.01, ^∗∗∗^ *P* < 0.001.

As PTEN was a target for miR-22 in NRK-52E cells, it was tempting to speculate that PTEN might mediate the regulatory effects of miR-22 on autophagy and the synthesis of collagen. To test this hypothesis, NRK-52E cells were transfected with a PTEN overexpression plasmid or empty plasmid for 24 h before being treated with NG or HG for another 48 h. Western blots showed that forced expression of PTEN could suppress AKT-mTOR signaling, promote autophagic flux, and inhibit the expression of Col IV both in NG- and HG-treated cells (Figure 6(a)). Importantly, overexpression of PTEN could effectively antagonize miR-22-induced autophagy suppression and the expression of Col IV (Figure 6(b)).

## 4. Discussion

Several studies have demonstrated that miR-22 inhibition could attenuate the level of liver and cardiac fibrosis [23, 24]. Recently, it has been reported that miR-22 is upregulated in the kidney of STZ-induced DN mice [25]. However, little was known about the role and specific mechanism of miR-22 on TIF. The major findings of this study were that miR-22 acted as an inducer of TIF by suppressing autophagy in renal tubular epithelial cells and thus promoted the development of DN. miR-22 suppressed autophagy and induced the expression of Col IV partially by targeting PTEN. Suppression of miR-22 may be an effective measure in restraining TIF.

Autophagy that generated by various harmful factors contributes to maintenance of cell homeostasis. HG exposure induced neurotoxicity, promoted myocardial injury, and reduced angiogenic properties of human umbilical vein endothelial cells while suppressing autophagy. The induction of autophagy could effectively antagonize the cytotoxic effects of HG [26–28]. Moreover, several studies have suggested that autophagy may be a protective factor that antagonizes TIF and prevents the progression of DN [3, 4]. Furthermore, an increasing number of studies have indicated that miRNAs play an important role in autophagy and fibrosis. However, the relationship among miR-22, autophagy, and TIF in DN remains unclear. Therefore, DN rats and cell models were established and applied to investigate the function of miR-22 in TIF and determine the underlying mechanism. In the present study, we first showed that miR-22 was associated with autophagy in DN rats and it could induce the synthesis of Col IV and *α*-SMA by suppressing autophagy in renal tubular epithelial cells. On the contrary, miR-22 inhibition suppressed Col IV and *α*-SMA expression and thus represents a promising therapeutic target for DN. Nevertheless, the exact mechanism by which miR-22 works remains unclear.

PTEN is known as a tumor suppressor but it also acted as an antifibrotic factor. Previous studies have demonstrated that PTEN was associated with renal fibrosis in DN [5, 6]. In addition, PTEN is a key activator of autophagy in various kinds of disease models or cell types by inhibiting the PI3K/AKT/mTOR signaling pathway. Thus, PTEN may play an important role in preventing the development of TIF by inducing autophagy in DN. Our data showed that PTEN could promote autophagic flux and inhibit Col IV expression by suppressing AKT-mTOR signaling both in NG- and HG-treated NRK-52E cells. This suggests that targeting PTEN may be an effective treatment for TIF. To date, only a few miRNAs were found to execute their biological functions in DN via targeting PTEN. Moreover, little is known about the effect of miRNAs on autophagy and TIF. Previous studies have indicated that PTEN was a potential target of miR-22 in cancer cells [29, 30]. However, whether PTEN mediated the biological effect of miR-22 in DN remained to be explored. Another key finding of the present study was that miR-22 suppressed autophagy and induced the expression of Col IV partially by targeting PTEN. Further research is needed to explore some of the other biological effects and targets of miR-22 in renal tubular epithelial cells as it would bring us a step closer to identifying the underlying mechanism in the pathogenesis of DN.

**Figure 7:**
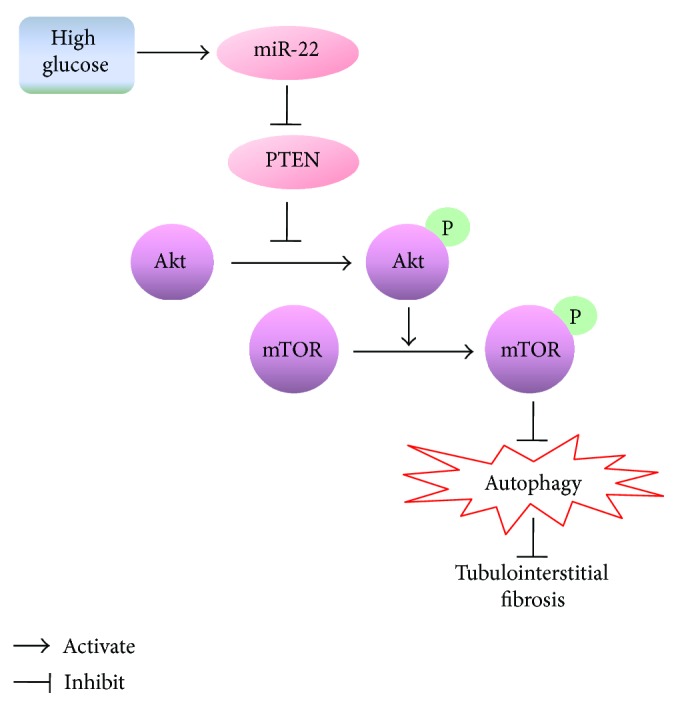
Principle signaling pathways involved in the effect of HG on autophagy and the expression of Col IV and *α*-SMA in NRK-52E cells. HG suppressed autophagy and in turn promoted Col IV and *α*-SMA expression via regulating PTEN/Akt/mTOR signaling pathway. By targeting PTEN, miR-22 mediated HG-inhibited autophagy and the synthesis of Col IV and *α*-SMA.

In summary, this study provides the novel finding that miR-22 was upregulated in renal tissues of DN rats and it exerted autophagy inhibition and profibrosis roles via targeting PTEN (Figure 7). Targeting miR-22 may be a novel and promising therapeutic approach in preventing the progression of DN.

## Figures and Tables

**Table 1:** The levels of kidney index (KI), blood glucose (BG), 24 h urine protein (24 h UP) in normal control (NC) group and diabetic nephropathy (DN) group (mean ± SEM. *n* = 10).

Group	KI (mg/g)	BG (mmol/l)	24 h UP (mg)
NC	7.70 ± 0.45	7.02 ± 1.39	39.64 ± 7.18
DN	11.86 ± 0.99^∗^	28.55 ± 4.20^∗^	261.75 ± 112.98^∗^

^∗^ *P* < 0.05 versus control group.

## Supplementary Materials

Supplementary Materials Figure 1: NRK-52E cells were treated with normal glucose, high glucose, or mannitol for 48 h; the protein levels of LC3-I, LC3-II, p62, and Col IV were detected by Western blot. ImageJ densitometric analysis of the protein levels; data represent mean ± SEM, *n* = 3, ^∗^ *P* < 0.05, ^∗∗^ *P* < 0.01, ns: not significant.
